# Preoperative characteristics of working-age patients undergoing total knee arthroplasty

**DOI:** 10.1371/journal.pone.0183550

**Published:** 2017-08-25

**Authors:** Tjerk H. Hylkema, Martin Stevens, Jan Van Beveren, Paul C. Rijk, Hans Peter van Jonbergen, Reinoud W. Brouwer, Sjoerd K. Bulstra, Sandra Brouwer

**Affiliations:** 1 Department of Orthopedics, University Medical Center Groningen, University of Groningen, Groningen, The Netherlands; 2 Department of Health Sciences, Division of Community and Occupational Medicine, University Medical Center Groningen, University of Groningen, Groningen, The Netherlands; 3 Department of Orthopedics, Röpcke-Zweers Hospital Hardenberg, Hardenberg, The Netherlands; 4 Department of Orthopedics, Medical Center Leeuwarden, Leeuwarden, The Netherlands; 5 Department of Orthopedics, Deventer Hospital, Deventer, The Netherlands; 6 Department of Orthopedics, Martini Hospital Groningen, Groningen, The Netherlands; Harvard Medical School/BIDMC, UNITED STATES

## Abstract

**Objective:**

Total Knee Arthroplasty (TKA) is performed more in working-age (<65 years) patients. Until now, research in this patient population has been conducted mainly among retired (≥65 years) patients. Aim of this study was therefore to describe demographic, physical, psychological and social characteristics of working TKA patients and to subsequently compare these characteristics with retired TKA patients and the general population.

**Methods:**

A cross-sectional analysis. Preoperative data of 152 working TKA patients was used. These data were compared with existing data of retired TKA patients in hospital registers and with normative values from literature on the general population. Demographic, physical, psychological and social (including work) characteristics were analyzed.

**Results:**

The majority (83.8%) of working TKA patients was overweight (42.6%) or obese (41.2%), a majority (72.4%) was dealing with two or more comorbidities, and most (90%) had few depressive symptoms. Mean physical activity level was 2950 minutes per week. Compared to the retired TKA population, working TKA patients perceived significantly more stiffness and better physical functioning and vitality, were more physically active, and perceived better mental health. Compared to the general population working TKA patients perceived worse physical functioning, worse physical health and better mental health, and worked fewer hours.

**Conclusion:**

This study shows that a majority of working TKA patients are overweight/obese, have multiple comorbidities, but are highly active in light-intensity activities and have few depressive symptoms. Working patients scored overall better on preoperative characteristics than retired patients, and except for physical activity scored overall worse than the general population.

## Introduction

Osteoarthritis (OA) is characterized as a chronic, progressive and inflammatory disease[1]. It is one of the most frequent causes of disability in Western populations [1]. In the Netherlands, approximately 594,000 people were dealing with OA of the knee in 2011 [2]. The risk of developing OA of the knee increases with age and overweight/obesity [1, 3]. The increasing numbers of ageing patients and patients with overweight/obesity stress a need for medical treatment for end-stage knee OA in Western societies that will further rise in the coming decades [1–7].

End-stage knee OA can be surgically treated with a Total Knee Arthroplasty (TKA) [8, 9]. Senior patients used to be primarily allocated to TKA. Prosthetic survivorship is currently longer and long-term results are known [10, 11]. The resulting trend is an increased number of TKA procedures in the retirement-age patients (≥65) age group (henceforth ‘‘retired patients”), as well as growing numbers of working-age OA patients under 65 (henceforth ‘‘working patients”) undergoing TKA [12–18].

As the number of working patients grows, studies about the characteristics of this patient population are gaining more attention. Previous studies have examined mainly health outcomes, e.g. utilization rates, postoperative outcomes, revision rates and alternative treatments [17, 19–21], yet it is also important to consider preoperative characteristics of working patients. Two recent studies have examined preoperative characteristics of younger patients. Keeney et al. [22] compared younger (<55 years) with older (65–75 years) patients, including demographics, physical activity and functioning levels before and after TKA. Results showed that in the younger group predominantly females were undergoing TKA, and more patients were obese and less active compared to older patients. Singh et al. [23] explored preoperative demographic, physical and psychological characteristics among TKA patients in different age groups. They found that among younger patients a number of individuals with a body mass index >40 was significantly higher and the prevalence of depression and anxiety increased sevenfold over 13 years’ time.

So far, studies that examine preoperative characteristics of working patients remain scarce. Existing studies survey only a few preoperative characteristics, lacking for example social and work characteristics. Moreover, working versus retired TKA patients have not yet been investigated specifically. This, along with a higher prevalence of working patients in the future, stresses the need to gain more insight into preoperative characteristics of working TKA patients. For physicians and other health professionals a better understanding of working patients in comparison to retired patients and the general population is useful during preoperative counseling and especially for discussing postoperative expectations (e.g. with respect to work). The primary objective of this study is therefore to describe the demographic, physical, psychological and social (including work) characteristics of working TKA patients. The second objective is to compare these characteristics of working patients with retired patients. At last, the third objective is to compare these characteristics with the general population.

## Methods

### Study design and subjects

Preoperative data of patients in the working-age group were used from the prospective cohort study ‘Work participation In Patients with Osteoarthritis’ (WIPO). In this cohort patients with knee osteoarthritis planned for TKA between March 2012 and July 2014 were included. Preoperative and postoperative data were gathered during a two-year follow-up. Inclusion criteria were primary or secondary knee OA and undergoing TKA, preoperative employment and age 18–63. The age of 63 was chosen in order to complete the two-year follow-up before the Dutch retirement age of 65 years. Exclusion criteria were insufficient knowledge of the Dutch language and having undergone joint arthroplasty in the previous six months. Four hospitals participated: University Medical Center Groningen (UMCG) (tertiary university hospital), Medical Center Leeuwarden (MCL) (large teaching hospital), Martini Hospital Groningen (MHG) (large teaching hospital) and Röpcke-Zweers Hospital Hardenberg (general hospital). The study was approved by the Medical Ethical Committee of University Medical Center Groningen (METc 2012.153) and in accordance with the 1964 Helsinki declaration and its later amendments or comparable ethical standards. Informed consent was considered obtained if the patient granted our request to participate in the study by returning a set of completed questionnaires. If patients did not want to participate in the study, they were requested to return a blank questionnaire. Patients were informed of this way of obtaining consent by an information letter. In this information letter they were also informed of the voluntary nature of the study, and that all data was processed anonymously. The Medical Ethical Committee specifically approved this consent procedure.

Preoperative data of retired TKA patients were gathered by using existing datasets of patients older than 65, where TKA was performed between 2008 and 2013 at one of the following hospitals: UMCG, Haga Hospital The Hague (large teaching hospital), MCL and Deventer Hospital (large teaching hospital). Data about the characteristics of the general population were collected by using normative or reference data found in the literature.

### Procedures

Available data of working TKA patients, retired TKA patients and the general population were gathered from different sources (see Table 1). For the working TKA patients preoperative data of 152 patients of the WIPO cohort were used (see Study design and subjects). Twenty-two (14%) were included from a tertiary hospital (UMCG) and 130 (86%) from general/large teaching hospitals (MHG, MCL, Röpcke-Zweers).

**Table 1:** Overview of the available data of different questionnaires of working TKA patients, retired TKA patients and the general population.

Patient characteristic	Instrument	Working TKA patients	Retired TKA patients	General population
Demographic	Age	X	X	
	Gender	X	X	
	Educational level	X		X
Physical	BMI^a^	X		X
	Comorbidity	X		
	SQUASH	X	X	X
	WOMAC^b^	X	X	X
	RAND-36	X	X	X
Psychological	PHQ-9^c^	X		X
	RAND-36	X	X	X
Social (including work)	RAND-36	X	X	X
	Actual working hours	X	n.a.	X
	Contractual working hours	X	n.a.	X
	Self-employed or employee	X	n.a.	X

^a^ = body mass index;

^b^ = Western Ontario and McMaster Osteoarthritis Index;

^c^ = Patient Health Questionnaire 9;

n.a. = not applicable;

X = available data

Data about the characteristics of the retired patients (n = 523) was gathered from existing databases of four hospitals. Haga Hospital provided physical activity data of n = 96 patients, Deventer hospital self-perceived physical functioning data of n = 202 patients, MCL health-related quality of life data of n = 51 patients, and UMCG provided data of n = 175 patients for all characteristics.

Normative or reference values of the general population were available for demographic, physical, psychological and social characteristics. Normative data from the literature on self-perceived physical functioning, health-related quality of life and depressive symptoms were categorized into age classes (S1–S3 Tables).

### Measures

All data for working TKA patients and retired TKA patients were gathered with paper-based surveys.

#### Demographic characteristics

Data were collected about age, gender and educational level (categorized into low, medium and high).

#### Physical characteristics

Body Mass Index (BMI): In order to assess BMI, height and weight were asked. BMI scores were categorized into underweight (<18.50), normal (18.50–24.99), overweight (25.00–30.00) and obese (>30.00). Reference data for the general population were obtained from Statistics Netherlands [24].

Comorbidity: Comorbidity was measured by using a 27-item chronic conditions questionnaire of Statistics Netherlands [25]. Number of comorbidities was categorized into having no, one or two, and more than two comorbidities. Comorbidity could not be compared with retired patients, and not all hospitals registered comorbidity. Reference data for the general population were not available.

Self-perceived physical functioning: To measure self-perceived physical functioning the Dutch version of the Western Ontario and McMaster Universities Arthritis Index (WOMAC) was used [26]. Using a Likert-scale, subjects rate themselves on multiple items grouped into three domains: pain (5 items), stiffness (2 items) and physical functioning (17 items). Each subscale is scored as a summation of items. The scores of the subscales make up the total score. The total score was recoded into a 100-point scale, with a higher score representing better physical functioning. The WOMAC has proven to be valid and reliable [27, 28]. Normative values of a general population were obtained from an Australian population [29].

Physical health: Physical health was measured with four subscales of the Dutch version of the RAND 36-item Health Survey, which measures health-related quality of life [30], i.e. physical functioning, vitality, bodily pain, and physical role functioning limitations. Subscores range is 0–100 and higher scores reflect better perception of physical health. The RAND-36 is a reliable and valid instrument [30]. Normative values of a general population were obtained from a Dutch population [31].

Physical activity level: Physical activity was measured with the SQUASH questionnaire, which measures habitual physical activity [32]. Respondents were asked how many days per week they performed physical activities, how many minutes per day, and how intense the activities were. The SQUASH includes questions on commuting activities, activities at work or school, and household and leisure-time activities [32]. The total score is reproduced as minutes per week [33]. The SQUASH has been tested on reliability and validity in the general population and in persons after THA [34]. To compare with the general population, two analyses were conducted. First, total activity time of working patients was compared with data of 273 healthy Dutch individuals under age 65 in a previous cohort from UMCG [35]. This was considered as the first normative value. The extent to which the working TKA patients complied with the Dutch guideline of 30 minutes moderate-intensity activity at least five days per week was considered as the second normative value [36].

#### Psychological characteristics

Depressive symptoms: The PHQ-9 is the depression module from the PRIME-MD instrument for common mental disorders, which scores each of the 9 DSM IV criteria from 0 (‘not at all’) to 3 (‘nearly every day’) [37]. Subjects were asked how often they have been bothered by each of the depressive symptoms over the last two weeks. PHQ-9 scores range from 0 to 27, scores of 9 or less indicate no depression, 10 to 14 moderate depression, 15 to 19 moderately severe depression and 20 to 27 severe depression[37]. The PHQ-9 has shown to be reliable and valid. Normative values of a general population were obtained from a German population [38].

Psychological functioning: The subscales mental health and emotional role functioning of the RAND-36 were used [31]. Scores range from 0 to 100 and higher scores reflect higher perceived mental health/better emotional role functioning. Normative values of a general population were obtained from a Dutch population [31].

#### Social characteristics

Social functioning: The subscale social functioning of the RAND-36 was used [31]. Scores range from 0 to 100 and higher scores reflect higher perceived social functioning. Normative values of a general population were obtained from a Dutch population [31].

Work: To assess work status, contractual working hours, actual number of working hours and a question about being self-employed or an employee were asked. As reference value in the Dutch population, mean contractual working hours from 2010 were derived from Statistics Netherlands [39]. The number of patients who were self-employed or employees was also compared with 2011 data from Statistics Netherlands [40, 41].

### Statistical analysis

Statistical analyses were performed using IBM SPSS, version 20. Missing data were addressed by adding the average of a scale or questionnaire, in conformity with questionnaire recommendations. Missing values regarding self-perceived physical functioning, physical health, physical activity, psychological functioning and social functioning were multiple imputed. Multiple imputation using predictive mean matching method was used. Data was imputed m = 20 times, so that the pooled results can be considered reliable[42, 43].

To answer the first objective of this study, the working TKA patients were first described on demographic, physical, psychological and social characteristics. To answer the second objective, they were compared with the retired TKA patients by means of Independent Samples T-tests or Mann-Whitney U-tests and Chi-square tests.

To answer the third objective of this study, two analyses were conducted to compare the working patients with the general population. Normative values derived from the literature were presented in different age classes (see S1–S3 Tables). In the first analysis the normative value of the age class correlating to the average age (55 years) was compared with the mean value of our cohort. As the age range of our cohort was between 28 and 63, we also did a second age-match analysis calculating the total number of working patients per age class who scored above or below the normative value. The comparison of working TKA patients with the normative values of the general population was tested with a one-sample T-test. A *P-*value < .05 was considered statistically significant.

## Results

### Characteristics of the working TKA patients

The physical, psychological and social characteristics of the working TKA patients are presented in Table 2. Mean age of the working patients was 55 (sd = 5.5), ranging from 28 to 63 years, and 59% was women. Working patients mainly had a low (34.7%) or secondary (46.3%) educational level. A majority (83.8%) was dealing with overweight (42.6%) or obesity (41.2%). Most patients (72.4%) reported two or more comorbidities. Mean physical activity performed was 2950 minutes (49 hours and 10 minutes) per week; most of the time was spent on work or household activities primarily of light and moderate intensity. Most working patients had no or few depressive symptoms. Lastly, most patients were employees and worked 31.3 hours per week.

**Table 2:** Demographic, physical, psychological and social characteristics of working TKA patients compared with retired TKA patients and the general population.

Characteristics	Working TKA patients (n = 152)	Retired TKA patients (n = 523)	p-value^d^	General population	p-value^e^
**DEMOGRAPHIC**					
**Age** (mean(sd) (range))	55(5.5) (28–63)	74(6.0) (65–91)		X	
**Gender** (no. (%))			**0.004**		
Male	65(44.2)	158(29.5)		X	
Female	87(59.2)	377(70.5)		X	
**Educational level** (%)	(n = 147)^a^				
Low	34.7	X		30	
Secondary	46.3	X		42	
High	19.0	X		28	
**PHYSICAL**					
**BMI**^b^ **(%)**	(n = 148)^a^				
normal	16.2	X		54.5	
overweight	42.6	X		33.7	
obese	41.2	X		11.8	
**Self-perceived PF**^f^ (mean(se))	(n = 152)	(n = 523)			
Physical functioning	47.7 (1.4)	46.0 (0.6)	0.219	84.4^c^	**<0.001**
Pain	41.8 (1.8)	44.3 (1.0)	0.206	84.8^c^	**<0.001**
Stiffness	41.2 (1.6)	45.8 (0.8)	**0.005**	78.1^c^	**<0.001**
Total	45.8 (1.3)	45.8 (0.5)	0.999	X	
**Physical health** (mean(se))	(n = 152)	(n = 523)			
Physical functioning	31.5 (1.3)	25.4 (1.3)	**0.001**	84.0^c^	**<0.001**
Vitality	60.1 (1.6)	56.2 (1.2)	**0.038**	68.6^c^	**<0.001**
Bodily pain	38.3 (1.6)	37.9 (1.3)	0.876	71.8^c^	**<0.001**
Physical role functioning	35.0 (3.1)	31.5 (3.3)	0.432	74.5^c^	**<0.001**
**Physical activity** (mean minutes/week(se))	(n = 152)	(n = 523)			
Activities to/from work	164.7(46.3)	n.a.		21.2(130.3)	**0.002**
Activities at work	1507.9 (68.9)	n.a.		349.0(805.3)	**<0.001**
Household activities	777.6 (71.2)	770.9 (29.9)	0.917	645.9(886.5)	0.071
Leisure-time activities	500.4 (54.7)	503.9 (42.3)	0.953	485.6(808.4)	0.753
sports activities	73.5 (11.9)	132.9 (215.9)	0.788	62.8(206.8)	0.229
Activity intensity					
light	2009.8 (93.9)	1110.4 (29.2)	**<0.001**	X	
moderate	744.3 (72.3)	355.2 (12.6)	**<0.001**	X	
vigorous	196.5 (42.7)	196.5 (10.8)	0.999	X	
Total minutes	2950.6 (108.6)	1662.2 (36.4)	**<0.001**	1501.6(1528.3)	**<0.001**
Satisfies Dutch norm (30 min. moderate activity 5–7 days/week)	69.7%	47.9%	**<0.001**	64.8%	
**PSYCHOLOGICAL**					
**Depressive symptoms**	(n = 151)				
Mean (mean(sd))	4.09 (3.8)	X	X	3.12(3.57)	**0.002**
No depression (n (%))	137 (90.7)	X	X	X	
Moderate depression (n (%))	13 (8.6)	X	X	X	
Moderately severe depression (n (%))	1 (0.7)	X	X	X	
Severe depression (n(%))	0	X	X	X	
**Psychological functioning** (mean(se))	(n = 152)	(n = 535)			
Mental health	78.3 (1.2)	69.7 (1.4)	**<0.001**	75.6^c^	**0.030**
Emotional role functioning	74.7 (3.2)	70.2 (3.5)	0.345	81.6^c^	**0.027**
**SOCIAL**					
**Social functioning** (mean(se))	(n = 151)	(n = 181)			
Social functioning	69.3(2.2)	71.7(2.1)	0.417	83.5^c^	**<0.001**
**Work**					
Actual working hours/week (mean(sd))	33.9(17.8)	n.a.	n.a.	X	
Contractual working hours/week (mean(sd))	31.3(16.1)	n.a.	n.a.	34.4^c^	**0.022**
Self-employed or employee (%)					
self-employed	13.2	n.a.	n.a.	14.2	
employee	80.9	n.a.	n.a.	72.1	
missing	5.9	n.a.	n.a.	X	

^a^ = n reduced due to missing data;

^b^ = body mass index;

^c^ = SD not available;

^d^ = *p*-value of comparison between working and retired TKA patients;

^e^ = *p* -value of comparison between working TKA patients and general population;

^f^ = self-perceived physical functioning;

X = no data available;

n.a. = not applicable; bold values represent significant values (p < .05).

### Working TKA patients compared to retired TKA patients

Table 2 presents the characteristics of the retired patients. Mean age was 74 (sd = 6.0), ranging from 65–91 years; 70% were women, in contrast to the working patient cohort (59%). Major differences between the working and retired groups were observed on physical characteristics. Working patients perceived more stiffness (p = 0.005), better physical functioning (p = 0.001) and better vitality (p = 0.038). The total amount of physical activity was significantly (p<0.001) different (2950 versus 1662 minutes per week). Working patients performed more light- and moderate-intensity activities than retired patients (p<0.001). More working patients met the Dutch guideline of 30 minutes moderate-intensity activity at least five days per week, compared to retired patients (69% versus 47%). Significant differences were also observed on psychological characteristics: working patients perceived better mental health (p<0.001). Scores on social functioning were generally similar in both groups.

### Working TKA patients compared to the general population

The normative and reference data of the general population are presented in Table 2. Working patients showed significantly (p<0.001) worse self-perceived physical functioning and significantly (p<0.001) worse physical health than the general population. Working TKA patients performed more activity (p<0.001) than the general population (2950 vs. 1501 minutes per week). Almost seventy percent of the working patients met the Dutch guideline of 30 minutes moderate activity 5–7 days per week, compared with 64% in the general population. The two groups also differed significantly on some psychological and social characteristics. Working patients reported more depressive symptoms (p = 0.002) and perceived better mental health (p = 0.026). However, working patients perceived worse emotional role functioning (p = 0.027), perceived worse social functioning (p<0.001) and worked fewer hours per week (p = 0.022).

The second analysis of the comparison between working patients and the general population showed the following results. For physical health, almost all patients (n = 149, 99.3%) scored worse on physical functioning; 109 (72.8%) patients perceived worse physical role functioning, 95 patients (63.3%) scored worse on vitality and 148 patients (98.0%) perceived more pain. For self-perceived physical functioning, 148 patients (96.1%) scored worse on physical functioning, 144 patients (95.4%) scored worse on the pain subscale and 141 (95.3%) patients scored worse on the stiffness subscale. For psychological factors, 48 (31.8%) patients scored worse on mental health, 49 (32.7%) patients scored worse on emotional role functioning and 93 (61.2%) patients had more depressive symptoms. Lastly, 95 (63.3%) patients perceived worse social functioning than the general population.

## Discussion

Main study findings are that working TKA patients have distinct preoperative characteristics: a majority of patients had overweight/obesity, most patients had multiple comorbidities, patients performed a high amount of light-intensity physical activity and a few patients were depressed. In comparison to retired patients and the general population, the results showed that working patients scored overall better on preoperative characteristics than retired patients and scored overall worse—except for physical activity level—than the general population.

The physical characteristics of the working patients are of interest to discuss. Working patients scored significantly worse on all physical characteristics (pain, physical functioning, vitality, stiffness and physical role functioning) compared to the general population, but scored better than retired patients on physical functioning and vitality. The finding that all characteristics were different between working TKA patients and the general population is not unexpected, as knee OA is a painful and highly disabling illness [1, 44]. The majority of the working patients were dealing with two or more comorbidities as well as with overweight or obesity. It is known that obesity, anxiety, depression and cardiovascular diseases are closely related to limitations in self-perceived physical functioning in patients with knee or hip osteoarthritis [45, 46]. Singh et al. found that, in particular, the number of cardiovascular diseases of TKA patients increased between 1993 and 2005 [23]. It is therefore important to assess these comorbidities, which are correlated to poor outcomes and to promote health.

The working patients were highly active during the week, considering the 2950 minutes of activity per week. It is questionable to what extent the high physical activity level is helpful to their health status though, as a majority was overweight/obese and perceived poor physical functioning. Seventy percent met the Dutch norm of 30 minutes moderate-intensity activity per day, but that is apparently insufficient to reach normal weight, therefore the focus for these patients should be on caloric intake. Healthcare professionals need to stimulate working patients to lower their caloric intake per day in order to lose weight [47]. Losing weight will improve physical functioning and result in better postoperative outcomes [47–50]. The finding that working patients performed twice more activity than retired patients and the general population can be mainly attributed to working patients’ activities at work of 1507 minutes per week. The rather large difference in mean minutes per week of work activities between working patients and the general population can be explained by age and employment status of the two study samples. The working patients had a mean age of 55 and were all employed, in contrast to the general population, which had an average age of 62.7 and was probably not all employed. Employment status was not an inclusion criteria though.

With respect to psychological characteristics, working patients generally had a relatively good mental health with no depressive symptoms. Although working patients dealt on average with significantly more depressive symptoms than the general population, however this was considered not clinically relevant as both are under the threshold of being depressed (>10 symptoms). Singh et al., observed a sevenfold increase in the prevalence of depression among younger (<50 years) TKA patients [23]. Social functioning did not differ between working and retired patients, but working patients perceived significantly worse social functioning compared to the general population. Such poor social functioning was observed in other studies of retired TKA patients [51, 52]. A lack of social support negatively influences social functioning [53].

Working patients worked significantly fewer hours than the general population. This is a common phenomenon in other patient groups dealing with musculoskeletal disorders such as low back pain or rheumatoid arthritis [54, 55]. An additional analysis showed that working patients had mainly physically demanding or a combination of physically and mentally demanding jobs. In a study of Hermans et al. it was found that physically demanding jobs performed by working knee OA patients were leading to significant productivity loss and sickness absence [56]. In that study patients with overweight/obesity and knee pain were absent from work more frequently. Patients in the present study were also dealing with these characteristics, and this may explain the finding that working patients worked fewer hours than the general population.

This study has several strengths and limitations. One of the strengths is that is gives a broader insight into the preoperative characteristics from a representative sample of Dutch TKA patients, compared to previous studies. We used data with preoperative characteristics of working TKA patients covering demographic, physical, psychological and social (including work) characteristics. A second strength is that we were able to compare the characteristics with data of retired TKA patients and the general population, which strengthened the interpretation and grading of the characteristics of working patients. However, there are also some limitations which should be taken into account.

One of the limitations is that the data of working and retired patients was provided by different hospitals. The majority of working patients were planned for TKA in general hospitals, in contrast with the data of retired patients, which were mainly derived from an academic hospital. While more severe knee OA patients are operated in academic hospitals, the cohort of retired patients may include higher comorbidity cases. Moreover, while data was lacking for some characteristics of retired patients (e.g comorbidity), not all characteristics could be compared with retired patients, which limited the interpretation of the data. A limitation of the normative values of the general population is that these values were derived from different countries. Comparisons of normative data between countries (despite them all being Western countries) are sensitive to bias by external factors such as legislation, disability compensation rates and health care [57, 58]. Another limitation is the use of self-administered recall questionnaires to assess preoperative characteristics. With self-reported questionnaires, it is known that overestimation can be an intrinsic property for aspects like physical activity. For that reason it is advised not to use self-reported questionnaires at the individual level, but at the group level, as was done in the present study [59, 60].

Results from the present study show that working TKA patients have distinct preoperative characteristics, including a majority with overweight/obesity, a high number of comorbidities, a large amount of light-intensity physical activity and few depressive symptoms. The high number of comorbidities and the overweight/obesity increases the chances of poor postoperative outcomes and delays recovery [49, 61, 62]. Comorbidities should therefore be assessed and treated, and more emphasis on promoting health should take place early on. The health-promoting focus needs to be on lowering the caloric intake of working patients in order to lose weight and thereby improve physical functioning [47]. Better physical functioning preoperatively will increase the chances of successful postoperative recovery [63–65]. Successful recovery is important for personal reasons but also from employers’ perspective, while return to work after surgery needs to be facilitated and preoperative loss of productivity at the workplace needs to be improved postoperatively to decrease indirect societal costs [66].

Further research is needed to identify the links between preoperative characteristics of working patients and postoperative outcomes. These relationships have been examined mainly in retired patients, but studies of working TKA patients are lacking. Moreover, studies that assess preoperative characteristics objectively are needed to prevent the overestimation of outcomes from self-reported questionnaires.

## Supporting information

S1 Table Mean values of the RAND-36 of a Dutch sample of healthy persons per age class [31]. (DOCX)

S2 Table Mean values for the PHQ-9 (Patient Health Questionnaire 9) of a German population per age class [38]. (DOCX)

S3 Table Mean values for the WOMAC (Western Ontario and McMaster Osteoarthritis Index) of an Australian population per age class[29]. (DOCX)
