# Supplementary material for: Preoperative characteristics of working-age patients undergoing total knee arthroplasty
Source: PLoS One. 2017 Aug 25;12(8):e0183550. doi: 10.1371/journal.pone.0183550 (PMC5571908; doi:10.1371/journal.pone.0183550)
Supplement: S1 Table — (DOCX) [file pone.0183550.s001.docx]

**Supporting information**

**S1 Table**

Mean values of the RAND-36 of a Dutch sample of healthy persons per age class [31]

| Age class in years: | 16-40 | 41-60 | 61-70 |
| --- | --- | --- | --- |
| Physical functioning | 93.1 | 84.0 | 71.7 |
| Physical role functioning | 86.4 | 74.5 | 67.3 |
| Bodily pain | 80.9 | 71.8 | 70.5 |
| Vitality | 70.7 | 68.6 | 67.7 |
| Social functioning | 87.8 | 83.5 | 82.0 |
| Emotional role functioning | 85.4 | 81.6 | 81.1 |
| Mental health | 78.7 | 75.6 | 76.9 |
