# Supplementary material for: Preoperative characteristics of working-age patients undergoing total knee arthroplasty
Source: PLoS One. 2017 Aug 25;12(8):e0183550. doi: 10.1371/journal.pone.0183550 (PMC5571908; doi:10.1371/journal.pone.0183550)
Supplement: S2 Table — (DOCX) [file pone.0183550.s002.docx]

**S2 Table**

Mean values for the PHQ-9 (Patient Health Questionnaire 9) of a German population per age class [38]

| Age class (years): | 25-34 | 35-44 | 45-54 | 55-64 |
| --- | --- | --- | --- | --- |
| Mean (sd) | 2.3 (3.2) | 2.6(3.5) | 2.8(3.5) | 3.2(3.5) |
