# Supplementary material for: Preoperative characteristics of working-age patients undergoing total knee arthroplasty
Source: PLoS One. 2017 Aug 25;12(8):e0183550. doi: 10.1371/journal.pone.0183550 (PMC5571908; doi:10.1371/journal.pone.0183550)
Supplement: S3 Table — (DOCX) [file pone.0183550.s003.docx]

**S3 Table**

Mean values for the WOMAC (Western Ontario and McMaster Osteoarthritis Index) of an Australian population per age class*

| Age class (years): | <35 | 35-39 | 40-44 | 45-49 | 50-54 | 55-59 | 60-64 |
| --- | --- | --- | --- | --- | --- | --- | --- |
| WOMAC pain | 92.4 | 90.7 | 89.9 | 89.1 | 86.4 | 84.8 | 84.4 |
| WOMAC function | 93.6 | 92 | 90.6 | 89.6 | 87.2 | 84.4 | 83.1 |
| WOMAC stiffness | 87.9 | 86.0 | 84.7 | 83.5 | 79.7 | 78.1 | 77.8 |

*Values are transformed to a 0-100 scale, whereby 100 indicates the best score [29].
